# Groundnut improvement: use of genetic and genomic tools

**DOI:** 10.3389/fpls.2013.00023

**Published:** 2013-02-25

**Authors:** Pasupuleti Janila, S. N. Nigam, Manish K. Pandey, P. Nagesh, Rajeev K. Varshney

**Affiliations:** ^1^International Crops Research Institute for the Semi-Arid TropicsPatancheru, Andhra Pradesh, India; ^2^Generation Challenge Programme, c/o Centro Internacional de Mejoramiento de Maíz y TrigoMexico DF, Mexico

**Keywords:** *Arachis hypogaea*, genetic variability, pedigree, disease resistance, phenotyping, QTLs, molecular breeding, genomic selection

## Abstract

Groundnut (*Arachis hypogaea* L.), a self-pollinated legume is an important crop cultivated in 24 million ha world over for extraction of edible oil and food uses. The kernels are rich in oil (48–50%) and protein (25–28%), and are source of several vitamins, minerals, antioxidants, biologically active polyphenols, flavonoids, and isoflavones. Improved varieties of groundnut with high yield potential were developed and released for cultivation world over. The improved varieties belong to different maturity durations and possess resistance to diseases, tolerance to drought, enhanced oil content, and improved quality traits for food uses. Conventional breeding procedures along with the tools for phenotyping were largely used in groundnut improvement programs. Mutations were used to induce variability and wide hybridization was attempted to tap variability from wild species. Low genetic variability has been a bottleneck for groundnut improvement. The vast potential of wild species, reservoir of new alleles remains under-utilized. Development of linkage maps of groundnut during the last decade was followed by identification of markers and quantitative trait loci for the target traits. Consequently, the last decade has witnessed the deployment of molecular breeding approaches to complement the ongoing groundnut improvement programs in USA, China, India, and Japan. The other potential advantages of molecular breeding are the feasibility to target multiple traits for improvement and provide tools to tap new alleles from wild species. The first groundnut variety developed through marker-assisted back-crossing is a root-knot nematode-resistant variety, NemaTAM in USA. The uptake of molecular breeding approaches in groundnut improvement programs by NARS partners in India and many African countries is slow or needs to be initiated in part due to inadequate infrastructure, high genotyping costs, and human capacities. Availability of draft genome sequence for diploid (AA and BB) and tetraploid, AABB genome species of* Arachis* in coming years is expected to bring low-cost genotyping to the groundnut community that will facilitate use of modern genetics and breeding approaches such as genome-wide association studies for trait mapping and genomic selection for crop improvement.

## INTRODUCTION

### ECONOMIC IMPORTANCE AND USES

**FIGURE 1:**
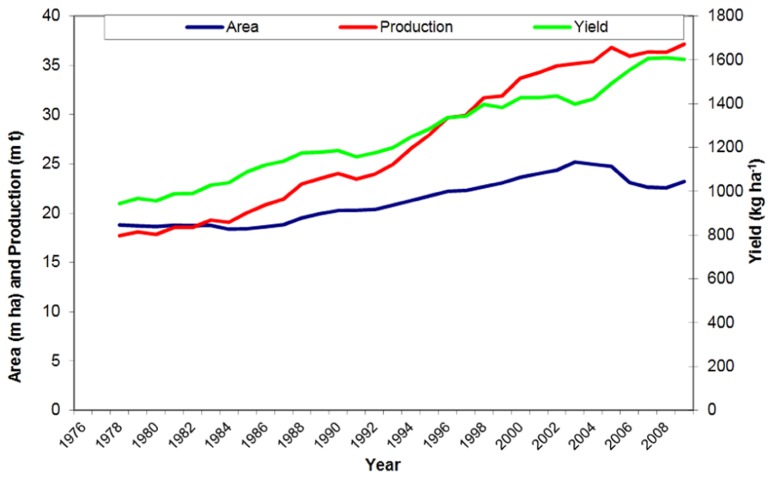
**Three-year moving center average for groundnut pod yield, production, and area harvested in world**.

Groundnut, also known as peanut, is an important oil, food, and feed legume crop grown in over 100 countries. It covered 24 million ha area worldwide with a total production of 38 million tons in 2010 (FAOSTAT, 2010). In the last decade (2000–2010), the global groundnut production increased marginally. The global annual increase in production was 0.4% which was due to both, an annual increase in yield by 0.1% and in area by 0.3% (**Figure 1**). The projected demand of groundnut in Asia alone by 2020 is expected to be 1.6 times more than the level of production in 2000 (Birthal et al., 2010). If the projected demands have to be met, the productivity and production of the crop has to increase at a much higher growth rate than the present one. Asia and Africa account for 95% of global groundnut area where it is cultivated under rainfed conditions with low inputs by resource poor farmers. Groundnut is a cash crop providing income and livelihoods to the farmer. It also contributes to nutrition of farm families through consumption of energy- and protein-rich groundnut kernels and provides nutritious fodder (haulms) to livestock. Thus groundnut cultivation contributes to the sustainability to mixed crop-livestock production systems, the most predominant system of the semi-arid areas.

Groundnut is valued as a rich source of energy contributed by oil (48–50%) and protein (25–28%) in the kernels. They provide 564 kcal of energy from 100 g of kernels (Jambunathan, 1991). In addition, the groundnut kernels contain many health enhancing nutrients such as minerals, antioxidants, and vitamins and are rich in mono-unsaturated fatty acids. They contain antioxidants like *p*-coumaric acid and resveratrol, Vitamin E, and many important B-complex groups of thiamin, pantothenic acid, vitamin B-6, folates, and niacin. Groundnut is a dietary source of biologically active polyphenols, flavonoids, and isoflavones. As they are highly nutritious, groundnut and products based on groundnut can be promoted as nutritional foods to fight energy, protein, and micronutrient malnutrition among the poor. Groundnut-based Plumpy’nut^1^, a ready to use therapeutic food, has helped save the lives of thousands of malnourished children in Niger (UNICEF, 2007).

Over 60% of global groundnut production is crushed for extraction of oil for edible and industrial uses, while 40% is consumed in food uses and others (such as seed for sowing the next season crop; Birthal et al., 2010). Groundnut oil is an excellent cooking medium because of its high smoking point (Singh and Diwakar, 1993). India, China, Myanmar, and Vietnam use groundnut oil for cooking purpose extensively. The cake obtained after extraction of oil is used in animal feed industry, in preparing enriched easily digestible food for children and aged persons, and as soil amendment. In Europe and North and South America about 75% of the production is used as food, while only 35% is used for the same purpose in Asia (Birthal et al., 2010). Peanut butter is the most popular groundnut product in the USA, Canada, and Australia. Groundnut seed can be consumed raw (non-heated), boiled, and roasted and also used to make confections and its flour to make baked products. The groundnut shells are used for making particle boards or used as fuel or filler in fertilizer and feed industry. Groundnut haulms constitute nutritious fodder for livestock. They contain protein (8–15%), lipids (1–3%), minerals (9–17%), and carbohydrate (38–45%) at levels higher than cereal fodder. The digestibility of nutrients in groundnut haulm is around 53% and that of crude protein 88% when fed to cattle. Haulms release energy up to 2337 cal kg^-1^ of dry matter. Being a legume crop, groundnut helps in improving soil health and fertility by leaving behind N_2_ and organic matter in the soil.

### TAXONOMY AND BIOLOGY

The cultivated groundnut (*Arachis hypogaea* L.), an annual herb belonging to the family Fabaceae (Leguminosae), is classified into two subspecies, subsp. *fastigiata* Waldron and subsp. *hypogaea* Krap. et. Rig. The subsp. *fastigiata* contains four botanical varieties, var. *vulgaris*, var. *fastigiata*, var. *peruviana*, and var. *aequatoriana*. The subsp. *hypogaea* contains two varieties, var. *hypogaea* and var. *hirsuta*. Each of these botanical types has different plant, pod, and seed characteristics (Krapovickas and Gregory, 1994). Groundnut is an allotetraploid (2n = 2x = 40) with “AA” and “BB” genomes. All species, except the cultivated species (*A. hypogaea* and *A. monticola*) in Section *Arachis*, and certain species in Section *Rhizomatosae*, are diploid (2n = 2x = 20). The diploid progenitors, *A. duranensis* and *A. ipaensis*, contributed “AA” and “BB” genomes, respectively, to the cultivated groundnut (Kochert et al., 1996). The phylogenetic analyses based on intron sequences and microsatellite markers also provide evidence for this hypothesis (Moretzsohn et al., 2012). A single hybridization event between the diploid progenitors followed by chromosome doubling (Kochert et al., 1996) about 3500 years ago lead to origin of cultivated groundnut. Southern Bolivia and Northern Argentina are thought to be center of origin of this crop (Gregory et al., 1980; Kochert et al., 1996). The center of diversity of the genus includes Western Brazil, Bolivia, Paraguay, and Northern Argentina (Gregory et al., 1980). *A. duranensis* occurs throughout the region, while *A. ipaensis* has only been found in Southern Bolivia. The genetic diversity of the genus is classified into four gene pools (Singh and Simpson, 1994): primary gene pool consisting of *A. hypogaea* and *A. monticola*, secondary consisting of diploid species from Section *Arachis* that are cross-compatible with *A. hypogaea*, tertiary consisting of species of the Section Procumbentes that are weakly cross-compatible with *A. hypogaea*, and the fourth gene pool consisting of the remaining wild *Arachis* species classified into seven other sections.

Groundnut is a self-pollinated crop with cleistogamous flowers, but natural hybridization can occur to small extent where bee activity is high (Nigam et al., 1983). Flowering begins 17–35 days after seedling emergence depending on the cultivar and environmental conditions. Flowers, simple or compound, are born in the axils of leaves and never at the same node as vegetative branch. One or more flowers may be present at a node. The stigma becomes receptive to pollen about 24-h before anthesis and remains so for about 12 h after anthesis, and the dehiscence of anthers takes place 2–3 h prior to opening of the flower in the morning. Fertilization occurs about 6 h after pollination. Depending upon the prevailing temperatures, the peg or gynophore carrying the ovary and fertilized ovule on its tip appears in 6–10 days and grows to enter the soil (positively geotropic) where it develops into pods. The tip orients itself horizontally away from tap root. Groundnut grows well in well-distributed rainfall of at least 500 mm. The growth and development is largely influenced by temperature in groundnut and the optimum air temperature is between 25 and 30°C. The nutritional requirement of groundnut is different as the pods develop in the soil. Calcium is an important nutrient required for pod and kernel development. It is unique to groundnuts that the pods directly absorb most of the calcium, and therefore calcium fertilizers are applied in the pod zone at the peak flowering stage to ensure its availability to the pods.

## TARGET TRAITS FOR GROUNDNUT IMPROVEMENT

The aim of groundnut breeding programs across the world is to develop new varieties that meet the requirements of grower, processor, and consumer. Thus the targeted traits for improvement in groundnut depend on the level of productivity achieved and consumers’ and industry requirements in a country. In the USA, where average productivity is high, the focus is more on improving food quality and flavor traits and freedom from mycotoxins in the produce. In the developing countries, where yields are generally low, the focus is more on removing yield barriers besides improving yield *per se*.

### YIELD PARAMETERS AND ADAPTATION

Yield and yield contributing parameters are the most widely targeted traits of groundnut improvement programs worldwide. Selection for yield *per se* has been the major basis for improving groundnut productivity in the world (Nigam et al., 1991), but gains from such selection have been low and slow due to large g × e interactions observed for these traits. The pod yield is a function of crop growth rate, duration of reproductive growth, and the fraction of crop growth rate partitioned toward pod yield. Therefore understanding physiology of yield is also essential to better target yield increase. The important yield contributing parameters are: pod yield per plant, number of pods per plant, shelling outturn, and 100-seed weight. Recently, physiological traits associated with yield (harvest index, transpiration-use-efficiency, etc.) are also receiving attention in breeding programs which have necessary infrastructure and resources. Other traits not having direct bearing on yield such as ease in shelling, ease in harvesting (peg strength), number of seeds per pod (for specific uses), reticulation, beak and constriction of pod, kernel shape and color, and blanching ability are also important considerations to satisfy farmers’, processors’, and market demands. For the development of dual purpose varieties, haulm yield becomes an important consideration in addition to pod yield. In addition to quantity of haulm produced, its quality determined by nitrogen content and digestibility are also important to breed dual purpose varieties. Crop duration and fresh seed dormancy in Spanish varieties are important adaptation traits. The maturity duration should match with the length of growing period (LGP; 90 to over 150 days) available at a given location and conditioned by the soil moisture availability and climatic conditions (mainly temperatures and sunshine hours). Early maturity is an important trait in groundnut as it enables escape from stress conditions such as drought and frost and to fit in multiple cropping systems. *In situ* germination, a consequence of lack of fresh seed dormancy leads to pod yield and quality loss in rainfed environments, particularly when rains coincide with the crop maturity stage.

### STRESS TOLERANCE/RESISTANCE

There is large gap between potential pod yield and the realized pod yield in most of the situations (Johansen and Nageswara Rao, 1996). Potential yield is defined as the maximum yield obtainable by the best genotypes in a specified agro-climatic environment when the known biotic and abiotic constraints are overcome. The yield gap in the groundnut grown under water limiting conditions in rainfed areas is further aggravated by incidence of a host of diseases and insect pests. Therefore, tolerance/resistance traits that offer protection against losses caused by biotic and abiotic stresses are important target traits. In addition to protection to yield, resistance/tolerance to stress factors enhances the quality (nutritional, visual appearance, sensory attributes, free from toxins, and post-harvest keeping quality) of both, pods and haulms that fetches better price in the market. However, studies have shown that high yield potential and high degree of resistance do not generally go together (Nigam et al., 1991), while the breeding programs target them together. Therefore, in most of the breeding programs a balance is struck between the yield potential and level of resistance to avoid any possible yield penalty. As a consequence, several varieties with high yield potential and moderate levels of resistance were bred and released for cultivation world over (see Some Accomplishments of Conventional Approaches). Drought and high temperature are the most important abiotic stresses that are widespread in groundnut-growing areas. Depending on the time of occurrence, drought can be characterized as early season, mid-season, and end-of-season drought. Mid- and end-of-season droughts are critical as they affect the pod yield and quality. Further, end-of-season drought predisposes pre-harvest *Aspergillus* infection in the field that affects quality of produce. Linked closely with drought is high temperature stress. Two key stages for heat stress in groundnut are: flowering including microsporogenesis (3–6 days before flowering), and fruit-set (Craufurd et al., 2002, 2003). The CGIAR’s climate change for agriculture and food security (CCAFS) research has shown that high temperature stress (above 30°C) will be widespread in East and Southern Africa, India, South East Asia, and Northern Latin America, which are important groundnut-growing areas. Thus, effort to breed varieties that can thrive and yield under both, drought and heat stress need to be intensified. With the increase in problem soils (saline and acid) across the cultivated lands of the world, breeding for tolerance to salinity and aluminum toxicity in acid soils are considered important target traits for groundnut improvement in some countries such as China.

Groundnut is attacked by several diseases caused by fungi. Late leaf spot (LLS) caused by *Phaeoisariopsis personata* (Berk. & Curt.) Van Arx, early leaf spot (ELS) caused by *Cercospora arachidicola* Hori and rust caused by *Puccinia arachidis* Spegazzini are among the major foliar fungal diseases worldwide. Aflatoxins are potent carcinogen produced by *Aspergillus* spp. infection in seed forcing several countries to have strict regimes in place on permissible levels of aflatoxins in their imports. *A. flavus* is predominant species in Asia and Africa, while *A. parasiticus* is in the USA. Stem and pod rot, caused by *Sclerotium rolfsii*, is potential threat to groundnut production in many warm, humid areas, especially where irrigated groundnut cultivation is expanding. Groundnut is also a host to several virus diseases, but only a few of them are economically important – groundnut rosette disease (GRD) in Africa, peanut bud necrosis disease (PBND) in India, tomato spotted wilt virus (TSWV) in the USA, peanut stripe potyvirus (PStV) in East and South East Asia, peanut stem necrosis disease (PSND) in pockets in Southern India, and peanut clump virus disease (PCVD) in West Africa (Nigam et al., 2012). Bacterial wilt, caused by *Ralstonia solanacearum*, is predominant among bacterial diseases of groundnut. Globally, nematodes cause 11.8% yield loss in groundnut. The root-knot nematodes, *Meloidogyne* spp. and the lesion nematodes, *Pratylenchus* spp. are important in groundnut (Sharma and McDonald, 1990). Aphids (*Aphis craccivora* Koch), several species of thrips (*Frankliniella schultzei*, *Thrips palmi*, and *F. fusca*), leaf miner (*Aproaerema modicella*), red hairy caterpillar (*Amsacta albistriga*), jassids (*Empoasca kerri* and *E. fabae*), and *Spodoptera* are the major insect pests in groundnut, among which aphids, thrips, and *Spodoptera* have worldwide distribution and cause serious damage (Wightman and Amin, 1988). In addition to causing yield losses, aphids and thrips are vectors of important virus diseases. Termites, white grubs, and storage pests also cause damage to groundnuts. Groundnut borer or weevil (*Caryedon serratus*) and rust-red flour beetle (*Tribolium castaneum*) are the major storage insect pests in groundnut. In most breeding programs across the world, breeding for resistance to diseases has received more attention than breeding for resistance to insect pest except when they are vector of viral disease. Another important reason for this is the availability of the resistant sources for diseases in cultivated and wild *Arachis* species.

### QUALITY PARAMETERS AND OTHER TRAITS

Quality includes both, physical and chemical attributes. Nutritional traits include oil, protein, sugar, iron and zinc content, fatty acid profile, and freedom from toxins, while the other quality parameters include visual and sensory attributes (consumer and trader preferred traits) and traits desirable in food/oil processing industries. Breeding for quality parameters in addition to yield enhances the economic returns to the farmers and other stakeholders along the value chain. Studies have shown that high oil content in groundnut is translated into economic benefits to both farmer and millers. Similarly, the produce with desirable traits for confectionary uses fetches higher price in the market because of its export value. Traits impacting on food and oil uses are also important; they include both quality and nutritional parameters. Depending on the nature of use, low oil and high protein contents (for food use), high oil content (for oil use), and high oleic/linoleic fatty acid ratio (for longer shelf-life) are important targeted traits in advanced breeding programs. The traits for confectionary uses in India are: greater proportion of sound mature kernels (SMK), flavor, 100 seed weight exceeding 55 g, >11% of sugar content, >24% of protein content, blanchability (>60%), and low oil content (<45%; Ramanathan, 2004). Seed mass is an important attribute to confectionary quality; however like yield and yield parameters, it is highly influenced by environment. The taste and sensory attributes of roasted groundnuts are associated with carbohydrate components of the kernel (Pattee et al., 2000). Seed color and shape and flavor are the other important confectionary attributes. Blanchability is removal of testa or seed coat (skin) from raw or roasted groundnuts and this attribute is of economic importance in processed groundnut food products, which include peanut butter, salted groundnuts, candies, and bakery products and groundnut flour. Groundnuts are rich source of several micronutrients, among which iron and zinc contents are important. Enhanced levels of iron and zinc are gaining importance with identification of biofortification (delivery of micronutrients *via* micronutrient-dense crops) as a cost-effective and sustainable approach to fight malnutrition among poor. Development of groundnut cultivars suitable to mechanization is important in developed countries and it is also becoming important in developing countries where human labor for agricultural operation is not available or expensive. Some of the traits for such cultivars include strong pegs, uniform maturity of pods, break resistant pods, non-protruding radicle, and less erect plants with pods near the tap root. Biological nitrogen fixation (BNF) is another important target trait in groundnut that can be improved through both, cultivar selection and *Rhizobium* strain improvement.

## GROUNDNUT IMPROVEMENT USING CONVENTIONAL APPROACHES

### GENETIC VARIABILITY AND GENETICS

The genetic variability in groundnut is low due to origin of the crop through a single hybridization event between two diploid species followed by a chromosome doubling and crossing barriers with wild diploid species (due of ploidy differences). The cultivated groundnut is an allotetraploid, while all wild *Arachis* species are diploid except *A. monticola* and certain species in Section Rhizomatosae. The low genetic variability for the traits of importance and polyploidy nature are a bottleneck to the groundnut improvement. The cultivated accession of *Arachis hypogaea* in the genebanks (repositories of plant genetic resources) and the advanced breeding lines in the breeding programs are the most frequently used sources of variability used as parents in hybridization. Groundnut genetic resources are available in the genebanks of ICRISAT, National Bureau of Plant Genetic Resources (NBPGR), and Directorate of Groundnut Research (DGR) in India; Oil Crops Research Institute of Chinese Academy of Agricultural Sciences and Crops Research Institute of Guangdong Academy of Agricultural Sciences in China; U. S. Department of Agriculture, Texas A&M University, and North Carolina State University in the USA; EMBRAPA - CENARGEN and the Instituto Agronômico de Campinas in Brazil; and Instituto Nacional de Tecnología Agropecuaria (INTA) and Instituto de Botánica del Nordeste (IBONE) in Argentina. Selection of an appropriate source that can be used as a parent in hybridization is challenging for two reasons; first adequate variability should be available to identify a source and second, the amenability of trait for improvement that is determined by the genetic nature of the trait. Much of the variability till remains poorly used in improvement programs. Nigam (2000) reported that only three disease resistant parents (J 11, NC Ac 17090, and PI 259747) appear in the parentage of the cultivars released in India. Ensuring genetic diversity in farmer’s fields is another important challenge to the breeder when limited number of sources is known.

Genetic variability for yield and yield attributes, resistance/tolerance to foliar fungal diseases (Singh et al., 2003), insect pests (Lynch, 1990), root-knot nematode (Simpson et al., 1993), traits for confectionary uses (Dwivedi and Nigam, 2005), oil content and quality (Upadhyaya et al., 2011), haulm yield and quality traits (Nigam and Blummel, 2010), and several other traits of economic importance were reported in literature (see **Table 1**). The germplasm accessions of cultivated groundnut in the gene banks are a vast repository of this variation. Wild *Arachis* species are also reported to possess desirable alleles for several economically important traits such as resistance to fungal and virus diseases, insect pests, and abiotic stress (Dwivedi et al., 2003, 2008; Rao et al., 2003; Kalyani et al., 2007; Nautiyal et al., 2008) and therefore can be potential sources for use in improvement of groundnut (Rao et al., 2003). However, a large proportion of cultivated and wild genetic resources of groundnut are yet to be studied and characterized for targeted and other traits of economic importance (see Pandey et al., 2012b for available collections of genetic resources). The concept of core and min-core collections was suggested to enable handling of the vast collections in gene banks and lay hands on appropriate sources. Core (representing 10% of total collection) and mini-core (representing 10% of core collection) collections were made for the groundnut genetic resources available in USDA/ARS (Holbrook et al., 1993; Holbrook and Dong, 2005), ICRISAT (Upadhyaya et al., 2002, 2003), and China (Jiang et al., 2008, 2010). Holbrook and Isleib (2001) observed that the resistance genes were cluster geographically and accessions with multiple disease resistance were most common in India, Mozambique, and Senegal.

**Table 1:** List of important traits in groundnut for which variability was reported in literature.

Trait	Reference
**Agronomic traits**
Yield and yield parameters	Nath and Alam (2002)
Pod characters	Swamy et al. (2006)
Seed dormancy	Wang et al. (2012)
Early maturity	Upadhyaya et al. (2006)
**Resistance to diseases and insect pests**
Early leaf spot (*Cercospora arachidicola* Hori)	Singh et al. (1997), Holbrook and Isleib (2001)
Late leaf spot [*Phaeoisariopsis personata* (Berk. & Curt.) Van Arx]	Singh et al. (1997, 2003), Holbrook and Isleib (2001), Li et al. (2012)
Leaf rust (*Puccinia arachidis* Spegazzini)	Singh et al. (1997, 2003)
Aflatoxin contamination (*Aspergillus *spp.)	Singh et al. (1997), Whitaker et al. (2004)
Stem and pod rot (*Sclerotium rolfsii*)	Singh et al. (1997), Gorbet et al. (2004)
Bacterial wilt resistance (*Ralstonia solanacearum*)	Singh et al. (1997)
Root-knot nematode (*Meloidogyne *spp.)	Holbrook and Isleib (2001)
Tomato spotted wilt virus (TSWV)	Li et al. (2012)
Groundnut rosette disease (GRD)	Subrahmanyam et al. (1998)
Kalahasty malady (*Tylenchorhynchus brevilineatus*)	Mehan et al. (1993)
Insect pests	Amin et al. (1985), Harish et al. (2012)
**Abiotic stress tolerance**
Drought tolerance	Upadhyaya (2005), Jogloy et al. (2009), Balota et al. (2012)
Heat	Ntare et al. (2001)
Cold	Upadhyaya et al. (2009)
Salinity	Singh et al. (2008)
Aluminum toxicity	Boshou et al. (2000)
Nitrogen fixation tolerant to soil drying	Devi et al. (2010)
**Quality parameters and others**
Nutritional quality	Wang et al. (2010), Upadhyaya et al. (2012)
Traits for confectionary uses	Dwivedi et al. (1994), Dwivedi and Nigam (2005)
Root hairs on roots and gynophores that enhance phosphorus uptake	Wissuwa and Ae (2001)
Phosphorus (P) uptake	Wissuwa and Ae (1998)

Understanding the nature of variability of the trait is important to select the breeding scheme to be employed for the improvement of the target trait. Reddy (1988) compiled the results of most of the reported genetic studies in groundnut up until 1986 in a book chapter. The results of genetic/inheritance studies on various traits in groundnut continue to appear in literature. Both, qualitative and quantitative inheritance is reported for the traits of importance. The qualitative traits, as expected, are governed by a few genes [rust (Paramasivam et al., 1990), root-knot nematode (Choi et al., 1999), rosette virus (Nigam and Bock, 1990), branching pattern (Pattanashetti et al., 2008), and high O/L ratio (Moore and Knauft, 1989)]. Both quantitative and qualitative inheritance is also reported for several traits by different workers [(resistance to LLS; Motagi et al., 2000; Dwivedi et al., 2002), fresh seed dormancy (Khalfaoui, 1991; Upadhyaya and Nigam, 1999)]. Most often, there is preponderance of additive genetic variance for quantitative traits [drought tolerance traits (Upadhyaya, 2005), and seed size (Venuprasad et al., 2011)]. Presence of dominance variance and epistasis variance due to allotetraploid nature of the crop are also reported for most of the quantitative traits, but these cannot be exploited in a self-pollinated crop such as groundnut. Inheritance of some of the traits of economic importance is not yet fully understood. In many other cases, not much is known about their inheritance pattern because of lack of effective phenotyping tools.

Studies on character association have resulted in identification of associated traits that result in maximizing gains through selection of both, target and its associated traits. In literature, several studies have demonstrated the utility of correlation analysis in groundnut based on plant and reproductive traits (Gomes and Lopes, 2005). Pod yield was reported to be positively associated with number and mass of mature pods per plant, number and mass of mature kernels per plant, shelling outturn, 100-seed mass, primary and secondary branches per plant, and harvest index. Thus, selection is exercised for these associated traits simultaneously with target trait. Sometimes character association studies have enabled the use of a robust, low-cost, and convenient surrogate trait as an alternative to a laborious trait for phenotyping and making selections. For instance, SPAD chlorophyll meter reading (SCMR) and specific leaf area (SLA) are highly correlated with water-use efficiency (WUE; Sheshashayee et al., 2006) and hence SPAD and SLA are used as the surrogate traits of WUE. At ICRISAT and several other groundnut breeding programs for drought tolerance, the trait-based approach wherein selections are based on surrogate traits of WUE, such as SPAD and SLA is adopted in combination with empirical approach to maximize the genetic gains.

The g × e has considerable influence on the progress of crop improvement and hence an important aspect for consideration. In groundnut, a majority of target traits of economic importance are polygenic and are highly influenced by environment that hinders the achievable genetic gains in breeding programs. Genetic analysis of yield revealed high influence of environment on pod yield (Zhang et al., 2011). High yielding cultivars with the least g × e interactions are normally desirable. However, when a cultivar is to be selected for a specific environment, the g × e interaction is desirable for maximizing production.

### BREEDING METHODS

The breeding methods used for self-pollinated crops are applied in groundnut breeding. They include mass selection, pedigree, bulk, single seed descent, and back-cross methods. Introduction and mass selections played an important role in the beginning, but later, hybridization followed by selection in segregating generations following different methods was predominantly practiced in breeding improved groundnut varieties. Emasculation and pollination procedures of hybridization are cumbersome and the success rate of making crosses is generally low, particularly when carried out by inexperienced hands. Another major challenge in groundnut breeding like in many other crops is the time (8 years or more) lag between hybridizing two parents and identification of an improved breeding line for release as variety.

Segregating populations derived from crossing two parents are most common in groundnut breeding. Nevertheless, multiple crossing systems, such as the double or convergent cross, to create adequate genotypic variability before selection (Wynne and Gregory, 1981) were also used. In groundnut, pedigree and bulk-pedigree methods of breeding are most frequently used to handle segregating populations derived from hybridization. Pedigree method allows breeders to practice selection of traits with high heritability, such as plant type, pod and seed size, and shape and testa color in early generations. Selection of quantitative traits such as yield and seed composition are made in later generations. Bulk-pedigree method is a modified method of bulk aimed at improving traits with low heritability traits (Wynne and Gregory, 1981). Single seed decent method is becoming popular as this has the advantage to save space and resources (Isleib et al., 1994). Back cross breeding is used only sparingly; however with the advent of molecular markers linked to the traits of interest and quantitative trait locus (QTL) identification and mapping, back-crossing is used frequently in breeding programs. This will be discussed in more details in molecular breeding (see Groundnut Improvement Using Genomic Tools). Population improvement procedures are not commonly used in groundnut because of difficulty in making large number of crosses required for population improvement. The genetic variability available in *A. hypogaea* genetic resources is being exploited through the above breeding procedures to develop improved groundnut cultivars. But utilization of variability from wild *Arachis* species has been limited due to reproductive isolation of the cultivated species, *A. hypogaea*, an allotetraploid, from its diploid wild species due to ploidy differences and sterility. Wide hybridization has been used to harness the potential alleles from wild species and this yielding fruits with regards to resistance to disease. Despite attempts the vast potential variability of wild *Arachis* species still remains locked. Mutation breeding, using both physical and chemical mutagens has also been extensively used in groundnut breeding to induce variability that has resulted in development of several improved varieties. By 1996 in China, 15% of new groundnut varieties were bred from induced mutants and they accounted for 19.5% of the cumulative cultivated area in the country (Qui et al., 1998).

Field designs play an important role from early stages of breeding that involve testing of a large number of genotypes for selection of few elite genotypes at later stages. Thus selection of an appropriate field designs is critical for efficient and optimal utilization of resources, while ensuring identification of best possible genetic combinations in breeding programs. Multi-environment testing is adopted for development of superior genotypes adapted to a wide range of environmental conditions. Participatory plant breeding has gained significance as it allows selection of genotypes for specific adaptation rather than for broad spatial adaptation (Ceccarelli et al., 2007; Vindhiyavarman et al., 2010) and it is particularly important in developing countries as it also paves way to adoption of new improved varieties. In countries like India, Vietnam, Indonesia, and African countries, lack of adoption of new varieties has been a major bottleneck to improved groundnut yields.

### PHENOTYPING TOOLS

Reliable and repeatable phenotyping remains the key to the success of any crop improvement program whether following conventional or molecular breeding approach. Several critical decisions in the process of breeding rely on results obtained from phenotyping. Phenotyping is important, first to identify a suitable source/donor for the trait, and second for selection and advancing the plants/progenies through the generations in the breeding cycles. Selecting a plant/progeny with desired combination of traits and rejecting the undesirable remains a challenging task in breeding programs as selections have to be exercised on a large number of plants/progenies with due consideration to a large number of traits simultaneously. Often, targeting multiple traits in breeding programs is limited by the challenges posed by simultaneous phenotyping for multiple traits. While in some cases it may not be feasible in others, though feasible, imposed screen for one trait may have confounding effect(s) on the other. For instance, our experience in foliar fungal disease screening nursery has shown that the rust pathogen, being obligate in nature, fails to establish and survive on leaf tissues that are already dead following leaf spot pathogen infection. As a consequence the plants infected with LLS pathogen will record low levels of rust infection and may get selected although the reason of low infection is other than resistance in this case. Another major pitfall in phenotyping is the proportion of chance escapes that get selected and hence the reliability and reproducibility are critical for a phenotyping procedure.

Phenotyping for qualitative traits, inherited by few major genes is relatively easy. Phenotyping tools are extensively used by breeders in collaboration with plant pathologists, physiologists, entomologists, biochemists, and food technologists for selection and advancement of breeding populations and evaluation of advanced breeding lines. The technique that is reproducible, robust, cost-effective, and non-destructive (for kernel oil content, etc.) is desirable for phenotyping as breeding programs in general have large number of segregating generations and advanced breeding lines to screen. Phenotyping for reaction to diseases includes screening under artificial inoculation in the laboratory, green house, and in field and under natural disease pressure at hot spot locations (Mayee and Munde, 1979; Foster et al., 1980; Nigam and Bock, 1990; Pande et al., 1994; Subrahmanyam et al., 1995; Shokes et al., 1998; Gorbet et al., 2004; Nigam et al., 2012). Either empirical approach that involves measuring the yield under imposed drought stress or salinity conditions or trait-based approach using surrogates or a combination of both are used for phenotyping abiotic stresses. Understanding of physiological mechanisms involved in abiotic stress tolerance enable use of associated physiological traits in breeding programs to enhance the efficiency of selection of desirable genotypes. Phenotyping tools for some traits such as *A. flavus* contamination in the field and reaction to soil-borne diseases need to be further developed to improve their repeatability. Similar is the case for phenotyping tools for reaction to insect pests. If surrogates for complex traits are found, they could be exploited for phenotyping of complex traits. Currently available phenotyping techniques for important target traits of groundnut breeding have been described by Janila and Nigam (2012) and are summarized in **Table 2**.

**Table 2:** Groundnut traits for which phenotyping methods are described in the literature.

Trait	Reference
**Abiotic stress tolerance**
Empirical approach for drought tolerance	Rao and Nigam (2003)
Transpiration efficiency	Ratnakumar et al. (2009)
SPAD chlorophyll meter reading (SCMR), specific leaf area (SLA), Carbon isotope discrimination [and thus water-use efficiency (WUE)]	Rao et al. (2001), Sheshashayee et al. (2003)
High temperature	Craufurd et al. (2002, 2003)
Salinity	Vadez et al. (2005), Singh et al. (2008)
Aluminum toxicity	Yang and Jing (2000), Pratap et al. (2002)
**Biotic stress tolerance/resistance**
A 1–9 scale for recording reaction of leaf spot and rust diseases in the field	Subrahmanyam et al. (1995)
Detached leaf technique for leaf spots and rust	Mayee and Munde (1979), Foster et al. (1980)
Aflatoxin contamination	Mehan and McDonald (1980), Mehan (1989), Holbrook et al. (1994)
Stem and pod rot	Pande et al. (1994), Shokes et al. (1998), Gorbet et al. (2004)
Groundnut rosette virus disease	Nigam and Bock (1990), Nigam et al. (2012)
Peanut bud necrosis disease	Pensuk et al. (2002), Nigam et al. (2012)
Tomato spotted wilt virus	Mandal et al. (2001), Nigam et al. (2012)
Peanut stripe virus disease	Middleton et al. (1988), Nigam et al. (2012)
Peanut stem necrosis disease	Nigam et al. (2012)
Bacterial diseases	Li and Tan (1984), Mehan et al. (1994)
Root-knot nematode	Holbrook et al. (1983)
Aphids	Zeyong et al. (1995)
Thrips	Dwivedi et al. (1995)
*Spodoptera litura*, leaf miner, and jassids	Ranga Rao and Wightman (1997)
**Quality traits**
Blanchability	Singh et al. (1996)
Seed coat color	Dwivedi and Nigam (2005)
Protein	Singh and Jambunathan (1980), Misra et al. (2000)
Oil content	Jambunathan et al. (1985), Misra et al. (2000)
Fatty acid	Phillips and Singleton (1981)
Iron and zinc	Sahrawat et al. (2002)
**Agronomic traits and others**
Maturity duration based on the concept of cumulative thermal time or degree days (CTT or °Cd), which is both, location and season neutral	Vasudeva Rao et al. (1992)
Haulm quality parameters	Nigam and Blummel (2010)
Biological nitrogen fixation efficiency of groundnut genotypes	Wynne et al. (1980), Nambiar et al. (1982), Nigam et al. (1985), Herdina and Silsbury (1990)

### SOME ACCOMPLISHMENTS OF CONVENTIONAL APPROACHES

Following the breeding procedures and using various techniques of phenotyping, several improved groundnut varieties were developed and released for cultivation world over. Since 1986, national partners in 36 countries have released 136 improved ICRISAT-bred varieties in their respective counties. Some of the varieties are released in more than one country. The national programs in India have released 189 improved groundnut varieties for cultivation in the country. The genetic gain for yield in India came from improvement in seed size, seed weight, and number of pods per plant (Ratnakumar et al., 2012). From their study, Reddy and Basu (1989) reported that improved varieties alone contributed to 30% yield increase in India since 1967. Details of some these varieties released in different countries follow.

JL 24, a popular short-duration groundnut cultivar in India, is a selection made from genotype EC 94943 introduced from Taiwan at the Oilseeds Research Station, Jalgaon, Maharashtra. It was released as JL 24 (Phule Pragati) in 1979 for cultivation in India (Patil et al., 1980). Subsequently, it was introduced to Africa and was released and commercially cultivated in several other countries (Chiyembekeza et al., 2001). It was also released in 1984/85 as Sinpadetha 2 in Myanmar and in 1992 as UPL Pn 10 in the Philippines. CG 7 (derived from USA 20 × TMV 10 cross), also known as ICGMS 42 or ICGV-SM 83708, is a high yielding Virginia bunch variety released in 1990. Recommended for cultivation in all groundnut-growing areas of Malawi, it is suitable for confectionery use and oil extraction. It is more tolerant to drought and much easier to harvest than Chalimbana, the most widely grown variety in Malawi (Subrahmanyam et al., 2000). Subsequently, it was also released in Tanzania as Mnanje in 2009, in Uganda as Serenut 1 R in 1999 and in Zambia as MGV 4 in 1990.

Following extensive screening of 5000 breeding lines and germplasm in wilt-sick plots in China and Indonesia, many genotypes with varying levels of resistance have been identified and using them bacterial wilt resistant cultivars were released in China, Indonesia, and other South East Asian countries that have offered protection against losses caused by the disease (Mehan et al., 1994; Wang et al., 2009). At ICRISAT, breeding for foliar fungal disease has resulted in development of several genotypes with significant levels of resistance to rust and LLS (Singh et al., 2003), which are either released for cultivation in several countries of Asia and Africa or have been used as parents in national breeding programs. However, the first few foliar diseases resistant releases such as Girnar 1 (derived from X 14-4-B-19-B × NC Ac 17090) in 1989 and ICG(FDRS) 10 (derived from X 14-4-B-19-B × PI 259747) in 1990 in India, in spite of their higher yields under disease epidemic conditions, did not find acceptance among the farmers due to their poor pod and kernel characteristics as a result of linkage drag.

Resistance to GRD was discovered in the late 1950s in local land races of Burkina Faso. Utilizing them, cultivars resistant to GRD, such as KH 149A, KH 241D, 69-101, RMP 12, RMP 91, and RG 1 were bred and released in the past in Africa. These cultivars are now used as sources of resistance as the land races were semi-erect and late maturing and several GRD resistant groundnut varieties have been released (Mayeux et al., 2003; Ndjeunga et al., 2003; Waliyar et al., 2007). Wide hybridization has been used to expand the gene pool by tapping alleles from wild species and several interspecific derivatives have been developed for use as donors of desirable traits or release as cultivars. One classical example of this is the release of two root-knot nematode-resistant groundnut cultivars (COAN) by the Texas Agricultural Experiment Station in 1999 after successful transfer of high level of nematode resistance into *A. hypogaea* from wild diploid species, *A. cardenasii* (Simpson and Starr, 2001). Varieties with multiple resistances were also bred following conventional methods and one such example is “Tifguard,” a groundnut variety bred for resistance to both root-knot nematode and TSWV (Holbrook et al., 2008). The screening methods for nematodes were useful to identify resistant source and subsequently use the source to breed genotypes with resistance to root-knot nematodes (Simpson et al., 2003) and Kalahasti malady (Tirupati 3; Mehan et al., 1993). GPBD 4, Spanish bunch groundnut genotype resistant to rust and LLS was released for cultivation in India (Gowda et al., 2002). ICGV 86855, one of the parents of GPBD 4, is an interspecific derivative between *A. hypogaea* × *A. cardenasii* resistant to rust and LLS.

Drought resistant genotypes of groundnut were developed by selecting high pod yield under imposed soil moisture stress, referred to as empirical approach (Branch and Kvien, 1992). Several drought tolerant varieties (55-437, GC 8-35, 55-21, 55-33, SRV 1-3, and SRV 1-96 among others) were developed in West Africa (Mayeux et al., 2003). Drought tolerant groundnut variety ICGV 91114, released in India for cultivation in drought-prone district Anantapur, which has world’s largest groundnut-growing area of about 800,000 ha in a district, has resulted in increase in net income by 36 and 30% reduction in year-to-year yield variability for farmers in the district (Birthal et al., 2011). In China, several new groundnut cultivars with improved productivity and some level of tolerance to aflatoxin contamination are extensively used in production in combination with integrated management approaches for aflatoxin management (Boshou et al., 2009). To increase breeding efficiency, studies on mechanisms of resistance to pre-harvest aflatoxin contamination were conducted and the most promising mechanisms identified were resistance to drought and resistance to the groundnut root-knot nematode (Holbrook et al., 2009). These approaches seem to be viable options for use in breeding in the absence of sources of resistance to aflatoxin contamination.

Varieties suitable for food uses were also released that include varieties for confectionary uses that have high seed mass, desirable seed shape, and flavor. The return of investment (ROI) was 133% for cultivation of large-seeded confectionary varieties, Asha (ICGV 86564) and Namnama (ICGV 90320) in the Philippines (PCARRD, 2009). Asha and Namnama were bred at ICRISAT. In 2002, they were introduced to Philippines and subsequently released in 2005 for Region 2 of Philippines through Bureau of Agricultural Research (BARI) and Philippines Council of Agriculture, Forestry and Natural Resources Research and Development (PCAARD). Another ICRISAT-bred variety, ICGV 00440 was released as Mallika in India. Induced mutagenesis along with recombination breeding at Bhabha Atomic Research Centre (BARC), Trombay, India succeeded in developing several large-seeded genotypes, of which TG 1, TKG 19A, Somnath (TGS 1), TPG 41, TLG 45, and TG 39 were released for cultivation to the Indian farmers (Badigannavar and Mondal, 2007). Groundnuts are bred for high oleic to linoleic ratio (O/L ratio) to improve the oil quality. Gorbet and Knauft (1997) registered the first high oleic line, SunOleic 95R, and it was followed by another variety, Hull with high O/L ratio and resistance to TSWV (Gorbet, 2007). In breeding programs targeting improvement of biological nitrogen fixation (BNF), high performing genotypes were identified at ICRISAT (ICRISAT, 1981) and North Carolina State University, Raleigh, USA (Wynne et al., 1980, 1983).

## GROUNDNUT IMPROVEMENT USING GENOMIC TOOLS

The conventional breeding approaches have been successful in development and release of several improved cultivars not just for increased yield but also for resistance/tolerance to biotic and abiotic stresses thus offering protection against losses and for consumer/trader preferred traits. The approaches were discussed highlighting some successes in Section “Groundnut Improvement Using Conventional Approaches.” However, the progress has been limited in improvement of some of the traits. It is possible to specifically target such traits for improvement, and also enhance the efficiency of overall breeding program through use of molecular breeding approaches. The following are the possible advantages of integration of molecular breeding approaches: (1) It provides tools to target traits of economic importance that remained poorly/not amenable to conventional breeding approaches in part due to their quantitative nature of inheritance and presence of high g × e. The identification of genomic regions, popularly called QTLs for quantitative traits is now routine, and thus it is possible to achieve genetic gains through selection for quantitative traits *via* selection of the genomic region harboring major QTLs for that trait; (2) Through molecular markers it is also possible to reduce the burden of linkage drag when the desirable traits are attempted to transfer from wild species or their derivatives into improved varieties. Systematic introgression of entire genome of a wild species into cultivated background is also possible by use of molecular markers, which is referred to as genome-wide introgression (GWI). Following this approach, it is possible to develop chromosome segment substitution lines (CSSLs) and advanced back-cross (AB)-QTL (AB-QTL) mapping populations which enable tapping of new alleles from wild species (Tanksley and Nelson, 1996). More details on these two approaches are given in Section “Emerging Genomics and Breeding Approaches”; (3) Optimum utilization of time and resources as several unwanted plants/progenies are rejected in early generations and also possibly at early growth stages of the plant based on the genotype data; (4) Phenotyping is not required in every generation as the plants/progenies can be advanced based on the genotypic data, and thus phenotyping can be postponed to later generations when non-segregating lines are derived. This results in drastic cut in the cost of phenotyping as well as increased efficiency of phenotyping when relatively less number of lines are needed to be evaluated; (5) Selection and advancing of the plants/progenies when done based on the genotyping data will eliminate the problem associated with chance failure of phenotype screen and chance escapes, which occur often with field screening. Molecular markers are unaffected by environmental fluctuations and hence, serve as reliable tags to track the target traits/genomic regions in breeding populations; (6) It is possible to improve an elite/popular cultivar for a target trait through marker-assisted back-crossing (MABC). Although back-crossing is a known method of breeding its utility in groundnut improvement remained meager till the advent of molecular technologies and there is renewed interest in this method of breeding through MABC; (7) It saves time significantly when recessive genes need to introgress/pyramid which is need of the hour in order to develop improved cultivars in shorter period of time; and (8) Gene pyramiding, i.e., targeting multiple traits is possible through molecular breeding as each of the target trait can be tracked simultaneously in the segregating populations though QTLs and/or markers.

Marker-assisted back-crossing has been the most preferred and result oriented molecular breeding approach for improving existing popular genotype for one or two traits and pyramiding of few genes/QTLs. Most importantly, MABC is now in routine use in development of near-isogenic lines (NILs) or CSSLs for genomics research. The limitation of this approach is its inability to handle multiple traits/QTLs/genes in one attempt due to requirement of large back-cross populations which becomes unmanageable (Ribaut and Hoisington, 1998; Varshney et al., 2012). Since, majority of the economically important traits are quantitative in nature and is governed by several genomics regions (genes/QTLs) distributed on plant genome and contribution of each is very small. Hence to improve such complex traits such as drought tolerance and seed yield wherein QTL analysis ends up with identification of several small-effect QTLs (Ravi et al., 2011; Gautami et al., 2012), a better molecular breeding approach called marker-assisted recurrent selection (MARS) has been proposed (Ribaut and Ragor, 2007) which can target more number of minor as well as major QTLs. MARS involves estimation of marker effects followed by two or three recombination cycles based on presence of marker alleles for small-effect QTLs (Bernardo and Charcosset, 2006; Eathington et al., 2007). Currently in groundnut, MABC is used for improvement of resistance to foliar fungal diseases, root-knot nematode and enhance oil quality for which linked markers for QTLs with high phenotypic effect were successfully identified. While, for improvement of drought tolerance in groundnut MARS was the suggested method of breeding as more than 100 main and epistatic effect QTLs were reported, it is not attempted due to lack of dense linkage maps in groundnut that limits the applicability of MARS (Ravi et al., 2011).

### MARKERS FOR TARGET TRAITS

Before the linkage maps were made available, bulk segregant analysis (BSA) was used to identify the linked markers for nematode resistance (Burrow et al., 1996; Garcia et al., 1996), aphid vector of GRD (Herselman et al., 2004), and yield and yield parameters (Selvaraj et al., 2009). But the approach of identifying markers for targets traits swiftly changed with the development of linkage maps in groundnut (see Pandey et al., 2012b). QTL analysis was used for identification of QTLs for several important traits such as drought tolerance related traits (Varshney et al., 2009; Ravi et al., 2011; Gautami et al., 2012), resistance to foliar disease (Khedikar et al., 2010; Mondal et al., 2012; Sujay et al., 2012), and nutritional quality traits (Chen et al., 2010; Chu et al., 2011; Sarvamangala et al., 2011) were reported. The availability of genomic resources such as molecular markers, genetic and physical maps, expressed sequence tags (ESTs), mutant resources, and functional genomics platforms that facilitate the identification of QTLs and discovery of genes associated with tolerance/resistance to abiotic and biotic stresses and agronomic traits was reviewed (Holbrook et al., 2011; Pandey et al., 2012b). Identification of target markers to traits of economic importance has enabled integration of molecular breeding in groundnut improvement as will be discussed in the following section.

### ONGOING MOLECULAR BREEDING ACTIVITIES FOR GROUNDNUT IMPROVEMENT

Despite several possibilities to overcome the bottlenecks of conventional breeding and some demonstrated successes in other crops including groundnut, the uptake of molecular breeding approaches in groundnut improvement programs in developed countries is contrasting with that in developing countries such as India and African countries. This may in be in part due to lack of infrastructure, high cost of genotyping, and human capacities available in the developing countries. The progress made when molecular breeding was integrated with conventional procedures in development of improved groundnut varieties was quite impressive. Holbrook et al. (2011) reviewed the impact of molecular genetic research, both genomic and transgenic tools on groundnut cultivar development. In this section we dwell upon some examples of its successful deployment. The first groundnut variety developed through integrated marker-assisted selection (MAS) in the USA is NemaTAM, a root-knot nematode-resistant variety (Simpson et al., 2003). However this was susceptible to TSWV. Subsequently a cultivar named, Tifguard, bred through conventional breeding method with resistance to both TSWV and root-knot nematode was released for cultivation in the country (Holbrook et al., 2008). Following marker-assisted breeding, “Tifguard High O/L” cultivar was developed through three rounds of accelerated back-crossing to pyramid nematode resistance and the trait for high oleic:linoleic acid (high O:L) ratio in seeds (Chu et al., 2011). In this MABC program, Tifguard was used as recurrent parent.

At ICRISAT, one of the goals of breeding program is to develop high yielding varieties with resistance to rust and LLS. After identification of a major QTL (QTL_rust_01) contributing up to 82.96% phenotypic variation for rust resistance, it was introgressed through MABC to improve three popular groundnut varieties (ICGV 91114, JL 24, and TAG 24) for rust resistance using GPBD 4 as a donor genotype. Several promising introgression lines with remarkable reduction in disease spread and other desirable agronomic traits have been selected for further multiplication and generation advancement (Pandey et al., 2012a). The promising genotypes with desirable yield potential and higher resistance to leaf rust could be released as improved varieties. MABC is also in progress for introgression of mutant fatty acid dehydrogenase (FAD) alleles on A and B genomes that govern high oleate trait (high oleic to linoleic acid ratio) that imparts high quality of oil benefiting both, consumers health and food processing industries (through enhanced shelf-life). The elite breeding lines with high oil content (>55%) and stable performance have been targeted for improvement of oil quality (Janila et al., 2012). In addition, gene pyramiding has also been initiated targeting one major QTL each for rust and LLS resistance after identification of major QTL for LLS resistance on AhXII that explains up to 62% of phenotypic variation from the same donor (Sujay et al., 2012).

Initiatives have been also taken at Chiba Prefectural Agriculture and Forestry Research Center in Japan, for marker-assisted introgression of mutant *FAD2* alleles into an elite cultivar “Nakateyutaka” using a breeding line “YI-0311” as donor which had an O/L ratio of 48 (Koilkonda et al., 2012). Nakateyutaka, a Virginia type is a leading variety in Japan and has normal O/L ratio. The *ahFAD2A* and *ahFAD2b* mutant alleles of the genotype YI-0311 were same as the previously reported mutational alleles found on high O/L groundnut genotypes (Jung et al., 2000) and used by Chu et al. (2011) in their MABC program. In addition to these ongoing molecular breeding activities the national programs for groundnut improvement in China and India have also initiated deployment of molecular breeding. The next decade may probably witness a good number of groundnut varieties developed though integrated molecular breeding approaches.

### TOOLS TO TAP ALLELES FROM WILD SPECIES

Genetic variability holds the key for the success of breeding program and groundnut has a twofold problem in this respect: first is the low genetic variability due its nature of origin, and second, the reproductive isolation from its wild diploid species due to ploidy differences and sterility. Wild *Arachis* species are known as repositories of several desirable alleles, however, wider use of wild species in breeding has been hampered by ploidy and sexual incompatibility barriers, by linkage drag, and historically, by a lack of the tools needed to conventionally confirm hybrid identities and track introgressed chromosomal segments (Bertioli et al., 2011). They remain under-utilized due to burden of linkage drag although the crossing barriers are to some extent overcome through techniques of wide hybridization. GWI and AB-QTL mapping are two important molecular marker-based approaches that enable enhanced utilization of alleles from wild species. GWI of a small genomic region from wild species while keeping the genetic background of the cultivated genotype is a good means to explore the largely untapped reservoir of useful alleles of interest in wild species. This is especially interesting in species like groundnut with narrow genetic base. This approach has been widely utilized for introgression of favorable QTLs for various traits in other crops such as tomato, rice, wheat, and barley (Fridman et al., 2004; Wang et al., 2005; Liu et al., 2006; Schmalenbach et al., 2009). AB-QTL mapping facilitates simultaneous discover of QTLs and development of elite genotypes (Tanksley and Nelson, 1996). AB-QTL mapping was used in tomato to breed an elite processing line (Tanksley et al., 1996). In groundnut, Foncéka et al. (2009) used AB-QTL approach to develop a genetic linkage map of wild genome introgression into cultivated background through utilization of synthetic amphidiploid between *A. duranensis* and *A. ipaensis*, and also derived CSSLs and AB populations. The CSSLs and AB populations facilitate characterization of different segments of genome of wild species contributing for resistance to foliar diseases and/or any other desirable trait. Once these different segments and their roles are determined, it is then possible to track them along the back-crosses using molecular markers for use in breeding programs. AB-QTL populations are also under development at ICRISAT. More recently, development of synthetic amphidiploids (Foncéka et al., 2009; Mallikarjuna et al., 2011) can facilitate better utilization of wild species in breeding programs as use of synthetic amphidiploids circumvent the crossing barriers between wild and cultivated species.

## EMERGING GENOMICS AND BREEDING APPROACHES

### GENOME SEQUENCE DATABASE

Because of large genome size and amphidiploid nature the genome and heavy costs associated, it was not possible earlier to initiate genome sequencing. However due to advances in next generation sequencing and coordination of large number of partners, Peanut Genome Project (PGP)^2^ has been initiated recently with specific goals for sequencing the groundnut genome and developing genomic resources for use in groundnut improvement programs (see Pandey et al., 2012b). It is expected that draft genome sequence and extensive genomic and transcriptome information will be available soon that will enable deployment of modern genotyping approaches such as genotyping-by-sequencing (GBS). GBS is expected to make genotyping costs cheaper and faster and accessible to a broad groundnut community. As a result, for trait mapping, genome-wide association studies (GWAS) will be in routine in coming years in groundnut breeding.

### GENOMIC SELECTION

Both MABC and MARS requires development of family mapping populations and identification of QTLs/marker effects before getting into the main stages of improvement programs. Further due to difficulty in handling polygenic traits and sometimes in development of good mapping populations, a better approach called “genomic selection (GS)” is fast emerging as a molecular breeding approach for crop improvement. Identification of superior lines with higher breeding value (genomic-estimated breeding values, GEBVs) in segregating breeding populations based on genome-wide marker profile data is the first step toward using this approach. To do so, a training population (TP) comprised of elite breeding lines for which multiple-season phenotyping data on agronomically important traits are available across environments is required for estimating GEBVs. Parental genotypes are then selected based on GEBVs and crosses are effected to develop candidate population (CP). In other words, CP is developed from the crosses made using the lines with best GEBVs in the TP as parents (Varshney et al., 2013). GS is now preferred over MABC and MARS for improving complex traits as GS has the advantage of selecting lines based on entire genome rather than one/few small segment of genome. It also enjoys the benefits of MABC and MARS by affecting selections based on genotype and prior of extensive phenotyping thus saving time and resources (Jannink et al., 2010). In order to exploit the recent advances in groundnut genomics to improve complex traits such as drought tolerance and seed yield, efforts have been initiated at ICRISAT to apply GS. In this direction, a TP has been developed that includes about 300 advanced breeding lines for which historical data on their performance have already been compiled.

## SUMMARY

The conventional breeding approaches have largely utilized the available genetic variability in cultivated groundnut and to some extent the variability trapped in wild *Arachis* species was also used to develop improved groundnut varieties. The breeding procedures used for self-pollinated crops are employed in groundnut improvement programs along with use of phenotyping tools. Identifying and assessing the nature of variability for target traits, utilizing the sources of variability as parents in hybridization, and advancing the best possible genotypes after selection are the key steps in the groundnut breeding programs. Pedigree, bulk-pedigree, and single seed decent methods are followed to handle segregating population after hybridizing two parents. Following conventional approaches, several improved groundnut varieties with high yield and tolerance/resistance to foliar fungal diseases, bacterial wilt, root-knot nematode, virus, rosette diseases, and drought were released for cultivation. The released varieties have a wide range of maturity duration, ranging between 90 and over 150 days required for cultivation in various growing regions with varying LGP. They belonged to different market types, *viz*, Spanish, Virginia, and Valencia and meet market uses (oil and food uses) and agro-climatic requirements.

The last decade has witnessed development of molecular marker linkage maps in groundnut that was followed by identification of markers and QTLs for target traits. This paved way for deployment of molecular tools in breeding program for efficient utilization of time and resources and improved efficiency of breeding. As a consequence, the extensive breeding programs are becoming intensive with the use of molecular breeding tools. Marker technologies offer approaches (GWI and AB-QTL mapping) that enable tapping of new alleles from wild *Arachis* species that remain under-utilized. Targeting multiple traits (gene pyramiding) is another important possibility of molecular breeding approaches. Groundnut breeding programs in USA, China, India, and Japan have already embarked the new technology to complement the ongoing breeding programs. “NemaTAM” is the first cultivar developed through molecular breeding for resistance to root-knot nematode. This was followed by “Tifguard High O/L” that has high O/L ratio and multiple resistances. At ICRISAT, MABC is underway to develop cultivars with rust resistance that are now in advance generations and also pyramid resistance to rust and LLS. MABC for improvement of oil quality is underway at USA, Japan, and India. The uptake of molecular breeding tools in groundnut breeding programs in developed countries is contrasting with developing countries like India and African countries, which is in part due to inadequate infrastructure, high genotyping costs, and inadequate human capacities in the later. On the other hand, more efficient genomic tools are under development, and this is expected to happen more rapidly once the draft genome sequence is available.

## Conflict of Interest Statement

The authors declare that the research was conducted in the absence of any commercial or financial relationships that could be construed as a potential conflict of interest.

## Footnotes

1 http://www.nutriset.fr/fr/nos-produits/produit-par-produit/plumpy-nut.html

2 http://www.peanutbioscience.com/peanutgenomeproject.html
